# Biochemical and Neurotransmitters Changes Associated with Tramadol in Streptozotocin-Induced Diabetes in Rats

**DOI:** 10.1155/2014/238780

**Published:** 2014-05-26

**Authors:** Essam Ezzeldin, Wafaa A. H. Souror, Toqa El-Nahhas, Abdel Nasser M. M. Soudi, Abdelaaty A. Shahat

**Affiliations:** ^1^Drug Bioavailability Laboratory, College of Pharmacy, King Saud University, P.O. Box 2457, Riyadh 11451, Saudi Arabia; ^2^National Organization for Drug Control and Research, P.O. Box 29 Cairo, Egypt; ^3^Pharmacology and Toxicology Department, Faculty of Pharmacy (Girls), Al Azhar University, Cairo, Egypt; ^4^Pharmacognosy Department, College of Pharmacy, King Saud University, P.O. Box 2457, Riyadh 11451, Saudi Arabia

## Abstract

The incidence of diabetes is increasing worldwide. Chronic neuropathic pain occurs in approximately 25% of diabetic patients. Tramadol, an atypical analgesic with a unique dual mechanism of action, is used in the management of painful diabetic neuropathy. It acts on monoamine transporters to inhibit the reuptake of norepinephrine (NE), serotonin (5-HT), and dopamine (DA). The purpose of this study was to evaluate the effects of diabetes on the brain neurotransmitter alterations induced by tramadol in rats, and to study the hepatic and renal toxicities of the drug. Eighty Sprague-Dawley rats were divided randomly into two sets: the normal set and the diabetic set. Diabetes was induced in rats. Tramadol was administered orally once daily for 28 days. The levels of DA, NE, and 5-HT in cerebral cortex, thalamus/hypothalamus, midbrain, and brainstem were evaluated in rats. In addition, the renal toxicity and histopathological effects of the drug were assessed. The induction of diabetes altered neurotransmitter levels. Oral administration of tramadol significantly decreased the neurotransmitter levels. Diabetes significantly altered the effects of tramadol in all brain regions. Tramadol affected function and histology of the liver and kidney. The clinical effects of tramadol in diabetic patients should be stressed.

## 1. Introduction

The incidence of diabetes has been increasing because of population growth, aging, urbanization, and increasing prevalence of obesity [1–3]. Globally, an estimated 150 million people are affected by diabetes, and this number is likely to reach at least 300 million by the year 2025 [4]. Moreover, tramadol (1RS, 2RS)-2-[(dimethylamino) methyl]-1-(3-methoxyphenyl)-cyclohexanol hydrochloride) is a potent centrally acting analgesic with affinity for opioid receptors in the micromolar range [5, 6]. Tramadol offers many advantages over conventional opioids and nonsteroidal anti-inflammatory drugs [7]. It is used mainly as an antidepressant for the action of drugs [8]. Moreover, it inhibits central monoaminergic uptake [9].

Tramadol was considered as an effective oral medication to relief pain in diabetic painful neuropathy [10, 11] and has dose dependent lowering effects on plasma glucose levels of diabetic rats [12]. Pharmacokinetic of tramadol has been influenced by diabetic condition [13].

The neurotoxicity of tramadol commonly manifests as generalized tonic-clonic seizures. Chronic use of tramadol in increasing doses causes neuronal degeneration in the rat brain, which probably contributes to cerebral dysfunction [14]. Tramadol also alters brain neurotransmitter levels [15].

The present study was designed to evaluate the changes in biochemical parameters and brain neurotransmitter level variations induced by diabetes and tramadol and their combination in rats.

## 2. Material and Methods

### 2.1. Animals

Male Sprague-Dawley rats weighing 180 ± 20 g were obtained from the animal house of the National Organization for Drug Control and Research (NODCAR), Egypt. The animals were grouped and housed in stainless steel cages under good laboratory conditions (temperature 25°C ± 2°C) with a dark/light cycle (12/12 h) for minimum of 7 days before the beginning of the experiments, to adjust to the new environment and to overcome the stress possibly incurred during transit. During this period, the animals had free access to a standard dry pellet diet and water ad libitum.

### 2.2. Induction of Diabetes

For the induction of diabetes, rats were fasted for 12 h before being administered a single intraperitoneal injection of 60 mg/kg of streptozotocin (STZ) [16]. STZ was prepared freshly in 0.05 M citrate buffer at pH 4.5. After 8 days of STZ injection, blood was obtained from the retroorbital vein and glucose levels were determined. Animals with glucose levels >250 mg/dL were considered as diabetic [17].

### 2.3. Experimental Design

Animals were divided into two sets, the normal set (nondiabetic) and the diabetic set. Each set was divided into a control group and three treated groups, with 10 animals each. Tramadol hydrochloride was dissolved in distilled water to 10 mg/mL/kg and administered orally either at 50, 75, or 100 mg/kg once daily for 30 days. The lowest dose of tramadol that was used in the present work was equivalent to the dose used by Munro [18] and middle dose comparable to dose used by Atici et al. [14].

At the end of the experiment (day 30 from the first day of tramadol administration), animals were scarified by cervical dislocation. The brain was excised rapidly. The brain was transferred to a dry, ice-cold glass plate and dissected into the following regions: cerebral cortex, thalamus/hypothalamus, midbrain, cerebellum, and brainstem. All tissues were patted dry and weighed. Brain tissue samples were stored at −80°C until analysis. Plasma was used for the determination of biochemical parameters. The liver and kidney were removed and stored in 10% formalin.

In all experiments conducted, the conditions were adjusted to decapitate the animal between 3:00 and 4:00 p.m. The animal utilization protocols were in accordance with the guidelines provided by the Experimental Animal Laboratory, and the study was approved by the Ethical Committee and Council of the General Division for Basic Medical Science, NODCAR (03-09-10).

### 2.4. Reagents

All chemicals used were of analytical grade. Reagents were stored in hard dark glass bottles with glass stoppers to avoid leaching of fluorescent contaminants: acidified* N*-butanol (0.85 mL of concentrated HCl was added to 1 L of* N*-butanol),* N*-heptane, hydrochloric acid (0.1 N and 10 N), sodium hydroxide (5 N), acetic acid (0.2 N and 5 N), iodine (0.1 N; 1.27 g of iodine was dissolved in 100 mL of absolute ethanol), EDTA (0.12 M; 0.1-M EDTA was dissolved in 1-N sodium acetate and the pH was adjusted to 7.0 with 5-N sodium hydroxide), NaHCO_3_ (0.033 M), and alkaline sulphite [25% (W/V), 0.5 g of anhydrous sodium sulphite was dissolved in 2 mL distilled water]. This solution was mixed well with 18 mL of 1 N NaOH just before use.* O*-phthalaldehyde (OPT; 4 mg and 10 mg) was dissolved in 10-N hydrochloric acid. Dopamine (DA) hydrochloride, norepinephrine (NE) hydrochloride, serotonin (5-HT), and hydrogen oxalate (obtained from Sigma-Aldrich, St. Louis, USA) were used in the experiments.

### 2.5. Extraction and Separation

The estimation of DA, NE, and 5-HT levels in the selected rat tissues was carried out according to the fluorometric method described by Ciarlone [19]. Each tissue sample was homogenized in 10 volumes of cold acidified* N*-butanol (3 mL) using a glass homogenizer. For plasma samples, 3 mL of acidified* N*-butanol was added to 0.3 mL of plasma.

Duplicate internal standard tubes were carried in parallel with the tissue homogenates. The internal standard was prepared by adding 0.4 mL of standard mixture (0.1 mL of each amine) to 9.6 mL of 0.1-N acetic acid. Aliquots of 0.2 mL of this solution were diluted into 0.3 mL of 0.2-N acetic acid, followed by the addition of 3 mL of acidified* N*-butanol.

The homogenate and internal standard tube were centrifuged at 2000 rpm for 5 min. Subsequently, 2.5 mL of the supernatant fluid was transferred to tubes, placed on a vortex mixer for 30 s, and the phases were separated by centrifugation at 2000 rpm for 5 min. 5-HT, NE, and DA were assayed in the aqueous phase.

### 2.6. Assay of DA and NE

The aqueous phase (1 mL) was transferred to a tube for the assay of DA and NE. External standards were prepared for NE and DA in duplicate in 0.2-N acetic acid and a total volume of 1.6 mL per tube. Acidified* N*-butanol (2.5 mL) and heptanes (5 mL) were added to the tube. All tubes were placed on a vortex mixer for 30 s and centrifuged at 2000 rpm for 5 min. The organic supernatant phase was discarded and 1 mL of the aqueous phase was transferred to a clean, dry test tube. EDTA reagent (0.2 mL) was added to all tubes (sample, internal standard, external standard, and reagent blank (1 mL of 0.2-N acetic acid)) and mixed. Subsequently, 0.1 mL of 0.1-N iodine was added and the solution was mixed again. Two minutes later, 0.2 mL of alkaline sulphite reagent was added and mixed. The tubes were allowed to stand exactly 2 min, followed by the addition of 0.2 mL of 5-N acetic acid and mixing. All tubes were placed in a boiling water bath for 2 min, cooled under tap water, and analyzed for NE fluorescence at excitation and emission wavelengths of 380 and 480 nm, respectively. All solutions were returned to their original test tubes, reheated in a boiling water bath for 5 min, cooled under tap water, and analyzed for DA fluorescence at excitation and emission wavelengths of 320 and 375 nm, respectively.

### 2.7. Assay of Serotonin

The aqueous phase (0.2 mL) was pipetted into test tubes. External standard was prepared in duplicate in 0.2-N acetic acid to a total volume of 0.2 mL. The blank consisted of 0.2 mL of 0.2-N acetic acid. To all tubes, 1.2 mL of 4 mg/dL OPT was added and mixed well. All tubes were placed in a boiling water bath for 10 min, cooled under tap water, and read in a spectrofluorometer at excitation and emission wave lengths of 295 and 355 nm, respectively.

### 2.8. Biochemical Analyses

Plasma alanine and aspartate transaminase (ALT and AST, resp.) activities were determined according to Reitman and Frankel [20]. Creatinine levels were evaluated using the quantitative kinetic colorimetric method using kits obtained from Roche Diagnostics (Mannheim, Germany). Blood glucose, urea, uric acid, triglycerides, and cholesterol levels were determined using urea kits of Diamond Diagnostic (Hanover, Germany). Plasma of ten animals was used to evaluate the abovementioned biochemical parameters. A Shimadzu UV 160 spectrophotometer was used to measure the values of theses parameters.

### 2.9. Histopathological Study

Livers and kidneys were fixed in 10% neutral-buffered formalin. The fixed specimens were washed, dehydrated, and embedded in paraffin wax. The tissues were sectioned at a thickness of 4–5 *μ*m and stained with hematoxylin and eosin (H&E) according to Bancroft and Stevens [21] as a routine procedure for histopathological examination.

### 2.10. Statistical Analysis

Results are presented as mean ± standard deviation (SD). Data were analyzed by one way analysis of variance (ANOVA). The lower limit for statistical analysis was defined as *P* < 0.05.

## 3. Results

Diabetes was induced in rats using STZ according to Kato et al. [16]. Tramadol was administered at doses of 50, 75, and 100 mg/kg. The lowest dose was comparable with the dose used by Munro [18]. The levels of monoamines (DA, 5-HT, and NE) were investigated in the cerebral cortex, thalamus/hypothalamus, midbrain, cerebellum, and brainstem in rats to evaluate the effects of diabetes on tramadol neurotoxicity. In addition, biochemical changes and liver and kidney histological alterations induced by diabetes and tramadol were investigated.

In diabetic rats, DA content was decreased in the cerebral cortex, midbrain, and brainstem regions, whereas it was increased in the cerebellum and thalamus/hypothalamus. Dopamine content was decreased in all regions examined in normal and diabetic rats treated with the different doses of tramadol. This decrease was dose dependent in the thalamus/hypothalamus, midbrain and cerebellum of normal rats and in the cerebellum and brain stem of diabetic rats (Table 1).

A decrease in NE levels was observed in all brain regions examined in the control group of the diabetic rat set compared with rats included in the control group of the normal set (Table 2). NE was also decreased in all regions examined in the brain of normal rats treated with tramadol. These decreases were dose dependent in all brain regions, with the exception of the brainstem. In diabetic rats, the decrease was significant in all regions examined in rats treated with tramadol, with the exception of the thalamus/hypothalamus, in which it was increased significantly after the administration of 50 mg/kg of the drug and decreased significantly after the administration of higher doses (75 and 100 mg/kg). Moreover, this decrease was dose dependent in the midbrain and cerebellum of diabetic animals (Table 2). All these changes were statistically significant compared with normal rats.

Diabetes affected 5-HT levels in different brain regions in different manners. Although diabetes increased 5-HT levels in the cerebral cortex, midbrain, and cerebellum, it decreased its levels in the brainstem and thalamus/hypothalamus region. Compared with the corresponding control group, the administration of tramadol to normal rats significantly decreased 5-HT levels in all brain regions examined. The diabetes-induced increment in 5-HT levels in the cerebellum and midbrain remained after the administration of different doses of tramadol compared with the corresponding group in the normal set (Table 3).

Diabetes itself increased significantly the levels of ALT, AST, creatinine, urea, triglycerides, cholesterol, and uric acid compared with the control group of normal animals. Tramadol at a dose of 50 mg/kg had no effect on the biochemical parameters examined in normal rats, whereas in diabetic rats, the levels of ALT, AST, creatinine, and urea were elevated significantly. The administration of tramadol at 75 mg/kg resulted in an increase in AST levels in nondiabetic rats and in AST, urea, creatinine, and triglyceride levels in diabetic rats. Tramadol at 100 mg/kg increased the levels of AST, ALT, urea, creatinine, and triglycerides in comparison to corresponding normal group. The administration of 100 mg/kg tramadol to diabetic animals resulted in an increase in the levels of AST, ALT, creatinine, urea, and triglycerides compared with the control group as well as the corresponding group in the normal set (Table 4).

**Figure 1:**
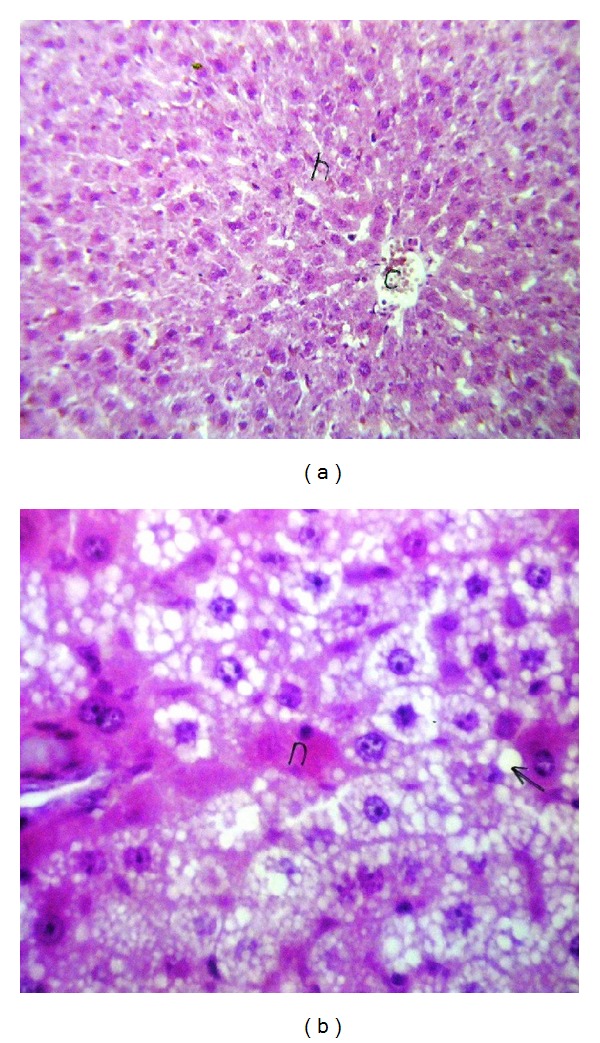
(a) Liver of normal rats. Normal histological structure of the central vein and surrounding hepatocytes, HE ×64. (b) Liver of diabetic rat. Some fatty changes with necrosis and necrobiosis were detected in the hepatocytes, HE ×80.

**Figure 2:**
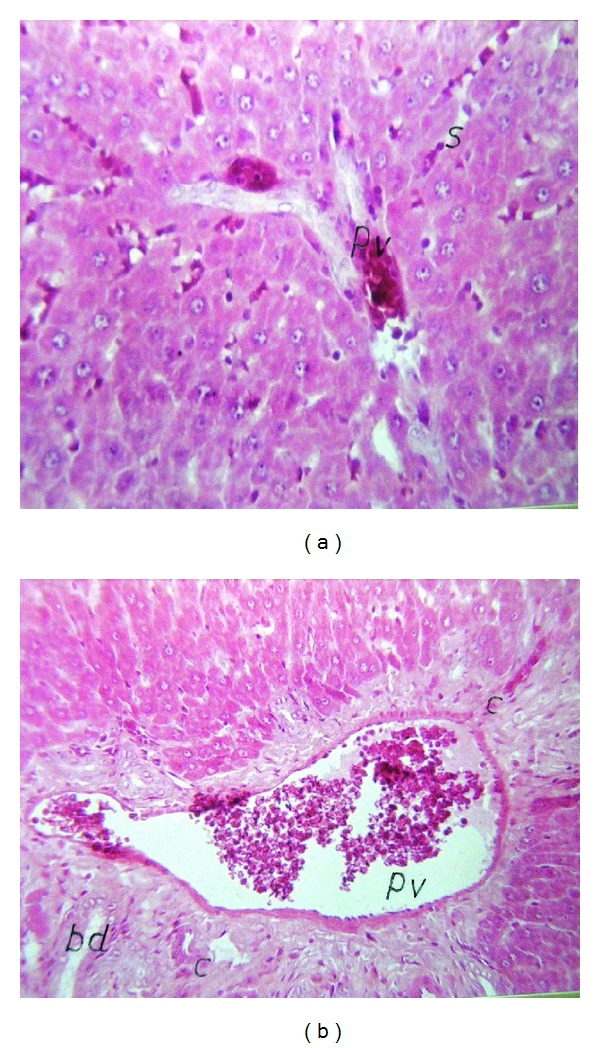
(a) Liver of rats treated with 50 mg/kg tramadol. Congestion in the portal veins (PV) and hepatic sinusoids, HE ×80. (b) Liver of diabetic rats treated with 50 mg/kg tramadol. Congestion in portal vein (pv) with collagen proliferation (c) in between the newly formed bile ductules (bd) at portal area, HE ×64.

**Figure 3:**
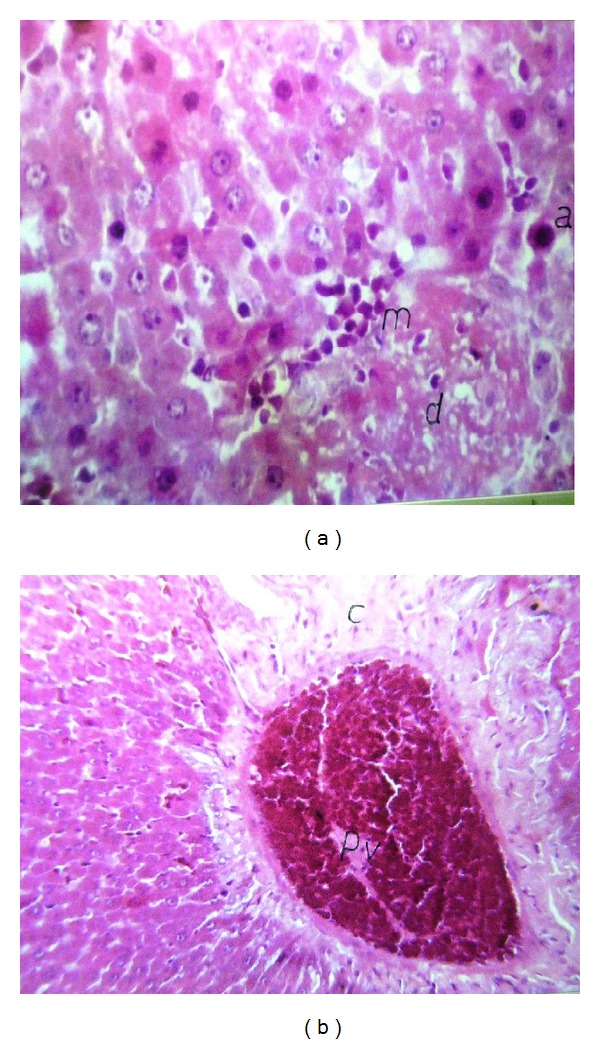
(a) Liver of normal rat treated with 75 mg/kg tramadol, Degenerative changes (d) and apoptosis (a) in the same hepatocytes with inflammatory cell infiltration (m). HE ×160. (b) Liver of diabetic rats treated with 75 mg/kg tramadol. Congestion in portal vein (PV) with collagen proliferation in portal area (C). HE ×64.

**Figure 4:**
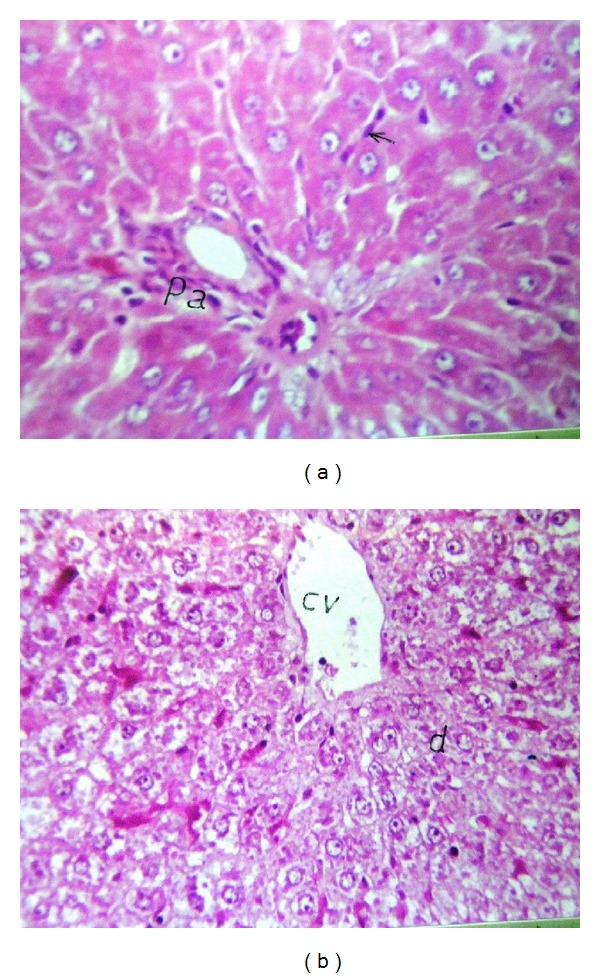
(a) Liver of normal rat treated with 100 mg/kg tramadol. Diffuse Kupffer cells proliferation (arrow) in between the some hepatocytes and surrounding the portal area (pa). HE ×80. (b) Liver of diabetic rat treated with 100 mg/kg tramadol. Dilatation in central vein (CV) with degeneration in the hepatocytes (d). HE ×80.

**Figure 5:**
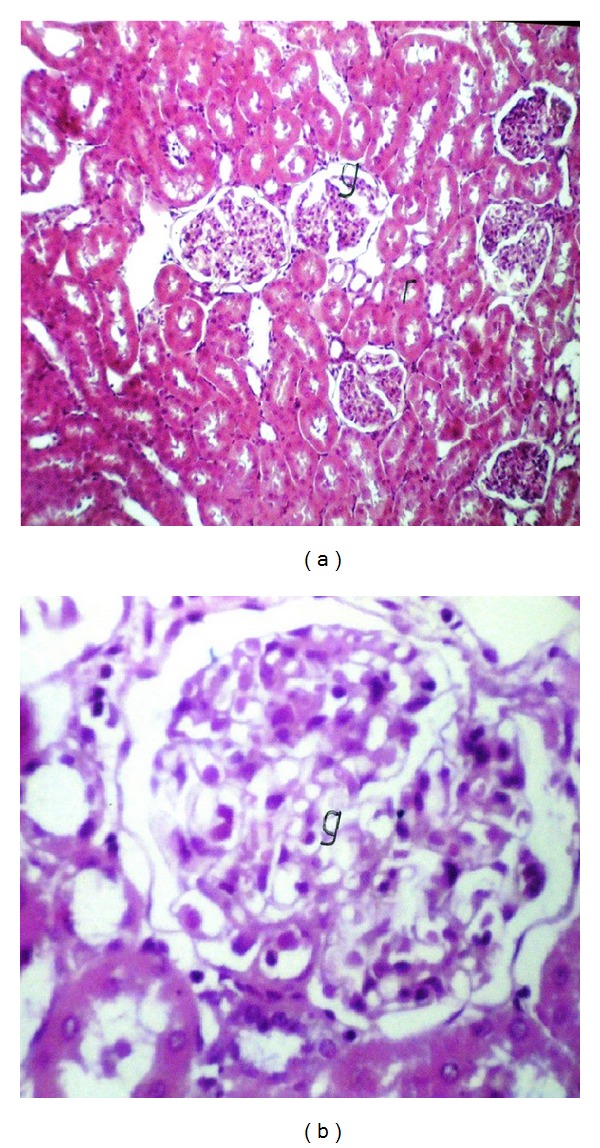
(a) Kidney of normal rats. No histopathological alteration, HE ×80. (b) Kidney of diabetic rats, glomerular tuft, swelling, and vacuolization in the lining endothelium associated with few inflammatory cells infiltration, HE ×160.

**Figure 6:**
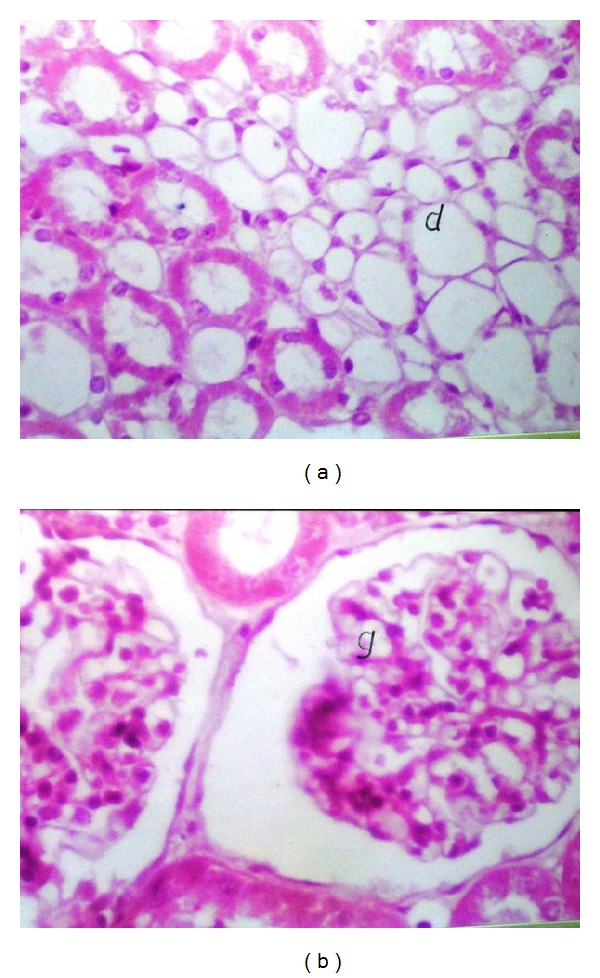
(a) Kidney of normal rats treated with 50 mg/kg tramadol. Degeneration and cystic dilatation (d) in tubules at corticomedullary portion, HE ×64. (b) Kidney of diabetic rat treated with 50 mg/kg tramadol. Swelling and vacuolization in the endothelial cells lining the tuft of glomeruli, HE ×160.

**Figure 7:**
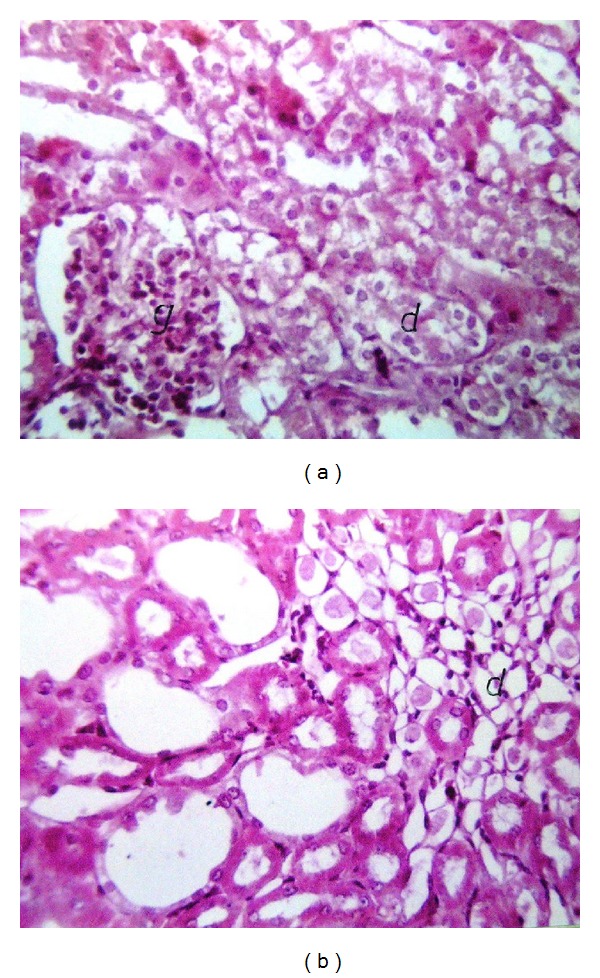
(a) Kidney of normal rat treated with 75 mg/kg tramadol. Degeneration and swelling (d) in the epithelial cells lining with the congestion in the tuft of the glomeruli (g) at the cortex, HE ×64. (b) Kidney of diabetic rat treated with 75 mg/kg tramadol. Renal cast formation in the tubular lumen (d) and cystic tubular dilatation at corticomedullary portion, HE ×64.

**Figure 8:**
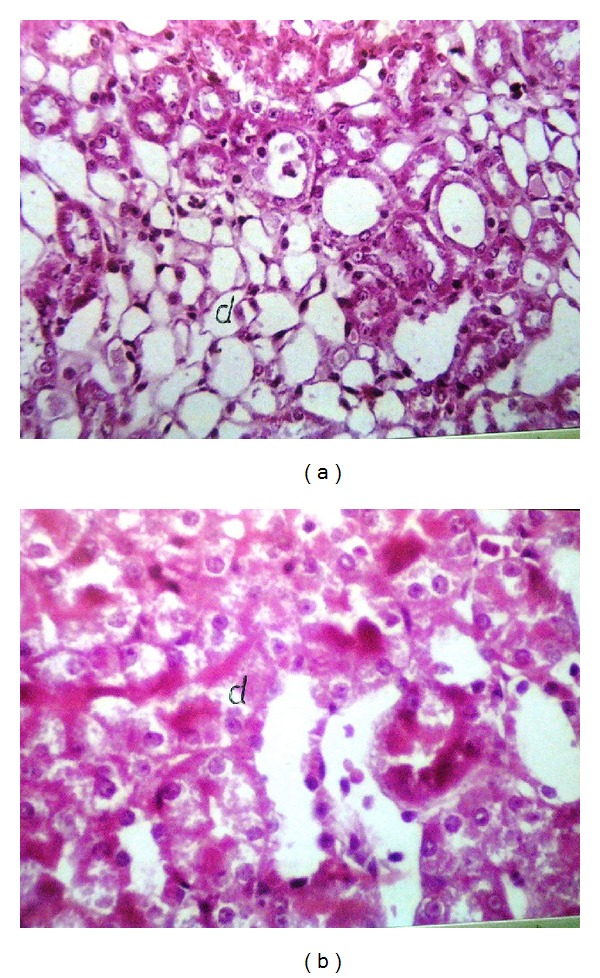
(a) Kidney of normal rat treated with 100 mg/kg tramadol. Degeneration and cystic dilatation in the tubules at the corticomedullary junction, HE ×64. (b) Kidney of diabetic rat treated with 100 mg/kg tramadol. Swelling and degeneration in the epithelial cells lining the tubular (d) at the cortex. HE ×80.

Liver (Figures 1–4) and kidney (Figures 5–8) samples of normal and diabetic rats treated with tramadol were investigated histologically. No histopathological structural alterations in the central vein and the hepatocytes surrounding it were observed in the control group of normally fed animals (Figure 1(a)). Fatty changes with necrosis and necrobiosis were detected in the liver of diabetic rats (Figure 1(b)), whereas hepatocyte architecture was affected by tramadol in normal and diabetic rats. The most striking histological findings in the liver were congestion (Figure 2(a)), dilated central and portal veins (Figures 2(b) and 3(b)), dilated hepatic sinusoids with collagen proliferation (Figures 2(a) and 2(b)), Kupffer cell proliferation between hepatocytes (Figure 4(a)), and apoptosis (Figure 3(a)) and degeneration of hepatocytes (Figures 3(a) and 4(b)). These alterations were dose dependent and were largely increased in the diabetic groups.

Histopathological examination of the kidney showed a normal histological structure in control rats of the normal set (Figure 5(a)); in contrast, in diabetic rats, histopathological investigation of the kidney showed glomerular swelling and vacuolization in the lining endothelium (Figure 5(b)). In tramadol-treated groups, the main histopathological findings in the kidney samples were vacuolization (Figures 5(a) and 5(b)), swelling of endothelial cells (Figures 5(b) and 6(b)), and association with degeneration in cells lining the tubule cortex (Figures 6(a) and 8(b)). Congestion in the tuft of the glomeruli at the cortex (Figures 6(b) and 7(a)), focal degeneration with cystic dilatation (Figures 6(a) and 8(a)), and renal cast formation in some tubules of the corticomedullary portion were observed (Figure 7(b)).

## 4. Discussion

The induction of diabetes by intraperitoneal administration of STZ (60 mg/kg) was similar to the work of Kato et al. [16]. Diabetes altered the levels of the brain neurotransmitters assayed. These changes were region specific. The specificity of regional changes was in agreement with Lackovic et al. [22] and may be caused by differences in gene expression in the different regions.

Diabetes significantly increased DA levels in thalamus/hypothalamus and cerebellum and decreased it in the cerebral cortex, midbrain, and brainstem. The results of the present work regarding DA decreases were in agreement with previous works of Shimomura et al. [23] and can be attributed to a reduction in DA synthesis and turnover in these brain regions.

A decrease in NE levels was observed in all brain regions of diabetic animals. The decrease in cerebellum NE in diabetic animals was in harmony with the results of Ramakrishnan et al. [24] and differed from the results obtained by Ramakrishnan and Namasivayam [25] and Hamdy and Taha [26]. These differences may be due to differences in animal strains or/and to the duration of diabetes.

Changes in 5-HT levels in diabetic animals varied among the regions examined. Compared with normal control rats, 5-HT levels were decreased in the thalamus/hypothalamus, cerebellum, and brainstem and increased in the cerebral cortex and midbrain. The increase in 5-HT levels was similar to the work of Ramakrishnan et al. [24] and was attributed to the increase in the density of the 5-HT2A receptor, whereas the decrease in 5-HT levels in diabetic rats was comparable to the previous work of Hamdy and Taha [26]. The decrease in 5-HT levels during diabetes may be due to a chronic anabolic deficit caused by a decrease in amino acids in the brain, with the consequent decrease in 5-HT synthesis [27]. These effects of diabetes can be attributed to the oxidative stress induced by STZ, as indicated by the increase in NO. It was shown that tramadol inhibited DA, NE, and 5-HT in the brain regions examined. In general, to some extent, these effects resemble in many aspects the action of the tricyclic antidepressant drugs [28].

The serotonin-decreasing effect of tramadol is in agreement with the results of Frink et al. [29] but is against those of Yalcin et al. [30]. The tramadol-decreasing effects can be attributed to its affinity for blocking 5-HT uptake [31].

Tramadol inhibited NE in all brain regions in normal and diabetic rats. The decreases in NE induced by tramadol may be due to its affinity to block NE uptake [28] and inhibit NE reuptake [32] as well as to the interaction between the tramadol metabolite O-desmethyltramadol and the NE receptor [29].

The biochemical changes induced by diabetes are in agreement with previous works on the increase of ALT, triglycerides [31, 33], uric acid [34, 35], and cholesterol [36] levels. These changes are compatible with the histopathological changes observed in liver and kidney tissues. The histopathological changes induced by diabetes may be due to disturbances in lipid accumulation [37].

## 5. Conclusion

Tramadol inhibited DA, NE, and 5-HT in the brain regions examined. Diabetes altered the levels of the brain neurotransmitters assayed. The cumulative effect of diabetes and tramadol on brain neurotransmitter (DA, NE, and 5-HT) levels has implications for diabetic patients who take tramadol. Future studies are recommended to explore the clinical effectiveness of the effects of diabetes mellitus on tramadol concerning liver and kidney histopathological and neurobehavioral toxicities in human.

## Figures and Tables

**Table 1:** Effects of tramadol on dopamine brain level in normal and diabetic rats.

	Dose (mg/kg)	Normal	Diabetic
Cerebral cortex	Control	0.802 ± 0.009	0.672 ± 0.007^+^
	50	0.632 ± 0.007*	0.605 ± 0.006^+∗^
	75	0.539 ± 0.005*	0.524 ± 0.005^+∗^
	100	0.560 ± 0.004*	0.526 ± 0.004^+∗^

Thalamus and hypothalamus	Control	1.333 ± 0.024	1.490 ± 0.017^+^
	50	1.191 ± 0.017*	1.244 ± 0.015^+∗^
	75	1.169 ± 0.013*	1.266 ± 0.012^+∗^
	100	0.888 ± 0.011*	1.32 ± 0.01^+∗^

Midbrain	Control	1.38 ± 0.022	1.178 ± 0.016^+^
	50	1.204 ± 0.016*	1.304 ± 0.013^+∗^
	75	1.031 ± 0.012*	1.007 ± 0.011^+∗^
	100	0.8773 ± 0.01*	0.805 ± 0.01^+∗^

Cerebellum	Control	0.87 ± 0.025	0.913 ± 0.017^+^
	50	0.77 ± 0.017*	0.850 ± 0.015^+∗^
	75	0.748 ± 0.013*	0.828 ± 0.012^+∗^
	100	0.711 ± 0.011*	0.790 ± 0.011^+∗^

Brain stem	Control	1.148 ± 0.023	1.006 ± 0.016^+^
	50	1.028 ± 0.016*	0.963 ± 0.014^+∗^
	75	1.021 ± 0.013*	0.956 ± 0.011^+∗^
	100	0.798 ± 0.011*	0.726 ± 0.010^+∗^

^+^Changes statistically significant in comparison to corresponding normal group, *P* < 0.001.

*Changes statistically significant in comparison to corresponding control group, *P* < 0.001.

**Table 2:** Effects of tramadol on norepinephrine brain level in normal and diabetic rats.

	Dose (mg/kg)	Normal	Diabetic
Cerebral cortex	Control	0.292 ± 0.007	0.277 ± 0.005^a^
	50	0.248 ± 0.005*	0.255 ± 0.004^a^
	75	0.242 ± 0.004*	0.288 ± 0.003^+^
	100	0.222 ± 0.003*	0.238 ± 0.003^+^

Thalamus and hypothalamus	Control	0.704 ± 0.014	0.605 ± 0.01
	50	0.649 ± 0.01*	0.623 ± 0.008^+∗^
	75	0.613 ± 0.007*	0.578 ± 0.007^+∗^
	100	0.579 ± 0.006*	0.562 ± 0.006^+∗^

Midbrain	Control	0.786 ± 0.001	0.686 ± 0.007
	50	0.574 ± 0.007*	0.628 ± 0.006^+^
	75	0.533 ± 0.006*	0.566 ± 0.005
	100	0.523 ± 0.005*	0.555 ± 0.004^+∗^

Cerebellum	Control	0.415 ± 0.01	0.401 ± 0.007^+^
	50	0.411 ± 0.007	0.415 ± 0.006^+∗^
	75	0.369 ± 0.006*	0.389 ± 0.005^+∗^
	100	0.356 ± 0.005*	0.374 ± 0.004^+∗^

Brain stem	Control	0.851 ± 0.014	0.697 ± 0.01^a^
	50	0.725 ± 0.01^s^	0.657 ± 0.008^+s^
	75	0.627 ± 0.008*	0.673 ± 0.007^+∗^
	100	0.647 ± 0.006^s^	0.632 ± 0.006^+∗^

^+^Changes statistically significant in comparison to corresponding normal group, *P* < 0.001.

^a^Changes statistically significant in comparison to corresponding normal group, *P* < 0.01.

*Changes statistically significant in comparison to corresponding control group, *P* < 0.001.

^s^Changes statistically significant in comparison to corresponding control group, *P* < 0.01.

**Table 3:** Effects of tramadol on serotonin brain level in normal and diabetic rats.

	Dose (mg/kg)	Normal	Diabetic
Cerebral cortex	Control	0.257 ± 0.006	0.267 ± 0.004^+^
	50	0.233 ± 0.004*	0.261 ± 0.004^+s^
	75	0.244 ± 0.003*	0.258 ± 0.003^+∗^
	100	0.248 ± 0.003^s^	0.221 ± 0.003^+∗^

Thalamus and hypothalamus	Control	0.732 ± 0.015	0.634 ± 0.01
	50	0.524 ± 0.01*	0.626 ± 0.009^+^
	75	0.596 ± 0.008*	0.584 ± 0.007^+∗^
	100	0.578 ± 0.006*	0.601 ± 0.006^+∗^

Midbrain	Control	0.662 ± 0.015	0.687 ± 0.01^+^
	50	0.593 ± 0.01*	0.662 ± 0.008^+∗^
	75	0.587 ± 0.008*	0.640 ± 0.007^+∗^
	100	0.562 ± 0.006*	0.648 ± 0.006^+∗^

Cerebellum	Control	0.219 ± 0.006	0.274 ± 0.004
	50	0.175 ± 0.004*	0.168 ± 0.003^+∗^
	75	0.178 ± 0.003*	0.210 ± 0.003^+∗^
	100	0.180 ± 0.003*	0.208 ± 0.002^+∗^

Brain stem	Control	0.573 ± 0.008	0.497 ± 0.060^a^
	50	0.444 ± 0.006*	0.464 ± 0.005^+∗^
	75	0.494 ± 0.004*	0.529 ± 0.004^+∗^
	100	0.495 ± 0.002*	0.499 ± 0.003^a∗^

^+^Changes statistically significant in comparison to corresponding normal group, *P* < 0.001.

^a^Changes statistically significant in comparison to corresponding normal group, *P* < 0.01.

*Changes statistically significant in comparison to corresponding control group, *P* < 0.001.

^s^Changes statistically significant in comparison to corresponding control group, *P* < 0.01.

**Table 4:** Effects of tramadol on liver and kidney functions in normal and diabetic rats.

Parameter	Doe (mg/Kg)							
	Control		50		75		100	
	*N*	STZ	*N*	STZ	*N*	STZ	*N*	STZ
AST (UL)	150.64 ± 12.2	170.96 ± 18.40^s^	159.28 ± 15.20	215.03 ± 20.60^+∗^	172.86 ± 6.80^s^	221.65 ± 22.06^+∗^	182.81 ± 15.8^x^	227.7 ± 22.0^+∗^
ALT (U/L)	31.92 ± 4.7	37.94 ± 3.50^b^	32.44 ± 16.50	46.16 ± 12.18	35.1 ± 1.50	45.33 ± 4.33^+a^	36.28 ± 1.40	48.27 ± 8.10^sa^
Creatinine	0.69 ± 0.08	0.9 ± 0.010^+^	0.77 ± 0.13	0.92 ± 0.013^ba^	0.7 ± 0.01	0.96 ± 0.04^+∗^	0.73 ± 1.20	0.97 ± 0.10
Urea	50.27 ± 16.62	61.12 ± 2.10^+^	48.07 ± 1.0	67.83 ± 2.59^+∗^	60.67 ± 4.30	68.75 ± 3.20*	61.0 ± 4.70	69.5 ± 2.80^+∗^
T.G.	36.46 ± 8.17	42.75 ± 2.6^+^	37.4 ± 10.96	58.9 ± 4.43^+∗^	38.00 ± 22.30	52.25 ± 1.40*	39.38 ± 17.60	54.00 ± 13.3
Cholesterol	67.71 ± 2.9	75.0 ± 3.70^b^	66.0 ± 18.70	76.75 ± 12.06	62.00 ± 7.50	76.5 ± 1.30^+^	62.88 ± 17.00	82.65 ± 8.30^s^
Uric acid	1.76 ± 0.062	2.16 ± 0.50	1.83 ± 0.064	2.65 ± 0.64^s^	1.87 ± 0.07	2.00 ± 0.60	1.93 ± 0.50	2.63 ± 0.60^s^

^+^Changes statistically significant compared to the corresponding normal group, *P* < 0.001.

^b^Changes statistically significant compared to the corresponding normal group, *P* < 0.01.

^s^Changes statistically significant compared to the corresponding normal group, *P* < 0.05.

*Changes statistically significant compared to the corresponding control group, *P* < 0.001.

^a^Changes statistically significant compared to the corresponding control group, *P* < 0.01.

^x^Changes statistically significant compared to the corresponding control group, *P* < 0.05.

*N*: normal rats, STZ: diabetic rats.
